# Intrinsic Capacity Assessment by a Mobile Geriatric Team During the Covid-19 Pandemic

**DOI:** 10.3389/fmed.2021.664681

**Published:** 2021-05-25

**Authors:** Davide Angioni, Camille Nicolay, Frédéric Vandergheynst, Robin Baré, Matteo Cesari, Sandra De Breucker

**Affiliations:** ^1^Hôpital Erasme, Service de Gériatrie, Université Libre de Bruxelles, Bruxelles, Belgium; ^2^CHU Toulouse, Service de Gériatrie, Toulouse, France; ^3^Hôpital Erasme, Service de Médecine Interne, Université Libre de Bruxelles, Bruxelles, Belgium; ^4^Geriatric Unit, Istituto di Ricovero e Cura a Carattere Scientifico (IRCCS) Istituti Clinici Scientifici Maugeri, Università degli Studi di Milano, Milan, Italy

**Keywords:** COVID-19, older adults, intrinsic capacity, decision-making, mobile geriatric team

## Abstract

In the autumn of 2020, the second wave of the COVID-19 pandemic hit Europe. In this context, because of the insufficient number of beds in geriatric COVID units, non-geriatric wards were confronted with a significant number of admissions of geriatric patients. In this perspective article, we describe the role of a mobile geriatric team in the framework of the COVID-19 pandemic and specifically how it assisted other specialists in the management of hospitalized geriatric patients by implementing a new approach: the systematic assessment and optimization of Intrinsic Capacity functions. For each patient, assessed by this consultative team, an individualized care plan, including an anticipated end-of-life decision-making process, was established. Intensity of care was most often not stated by considering chronological age but rather the comorbidity burden, the frailty status, and the patient's wishes. Further studies are needed to determine if this mobile geriatric team approach was beneficial in terms of mortality, length of stay, or functional, psychological, and cognitive outcomes in COVID-19 geriatric patients.

## Introduction

In the early autumn of 2020, the second wave of the COVID-19 pandemic hit Europe and the number of hospitalizations rapidly increased in several European countries (1). In October 2020, with about 8,500 new cases per day (considering symptomatic as well as asymptomatic patients) for a population of 11 million inhabitants^1^, Belgium neared a “coronavirus tsunami.” Age is one of the most critical risk factors for infection and negative outcomes of SARS-CoV-2 (2, 3), thus characterizing a “gero-pandemic” (4). Due to the lack of available geriatric COVID beds, non-geriatric wards were faced with countless hospitalizations of patients with a geriatric profile.

Geriatric patients present specific characteristics like comorbidity, polypharmacy, and physical frailty, making their management challenging for healthcare providers without geriatric training (5, 6). In order to capture the composite of older adult functions in a holistic way, the concept of intrinsic capacity (IC) was introduced in 2015 by the World Health Organization (7). Intrinsic capacity is defined as the composite of all the physical and mental capacities of an individual. Five domains are targeted: cognition, mobility, vitality, mood, and sensory domain (8).

The mobile geriatric team (MGT), initially described in the early eighties and later implemented in several countries, is a consultative team aimed to offer a multidisciplinary geriatric approach to older patients with a geriatric profile admitted in non-geriatric wards. The MGT, referred also in the literature as an inpatient geriatric consultation team or geriatric liaison team, is composed of nurses, occupational therapists, psychologists, speech therapists, dietitians, social workers, and physiotherapists, coordinated by one or more geriatricians (9). The early intervention of an MGT was shown to reduce the length of stay of geriatric inpatients (10) and was associated with a lower mortality rate and less functional decline after hospital discharge (11–13).

In this perspective article, we describe the role of a mobile geriatric team in the context of the emergency situation of the COVID-19 pandemic and specifically how it assisted other specialists in the management of geriatric patients by implementing a new approach: the systematic assessment and optimization of intrinsic capacity functions.

## The Mobile Geriatric Team During The COVID-19 Pandemic

**Figure 1:**
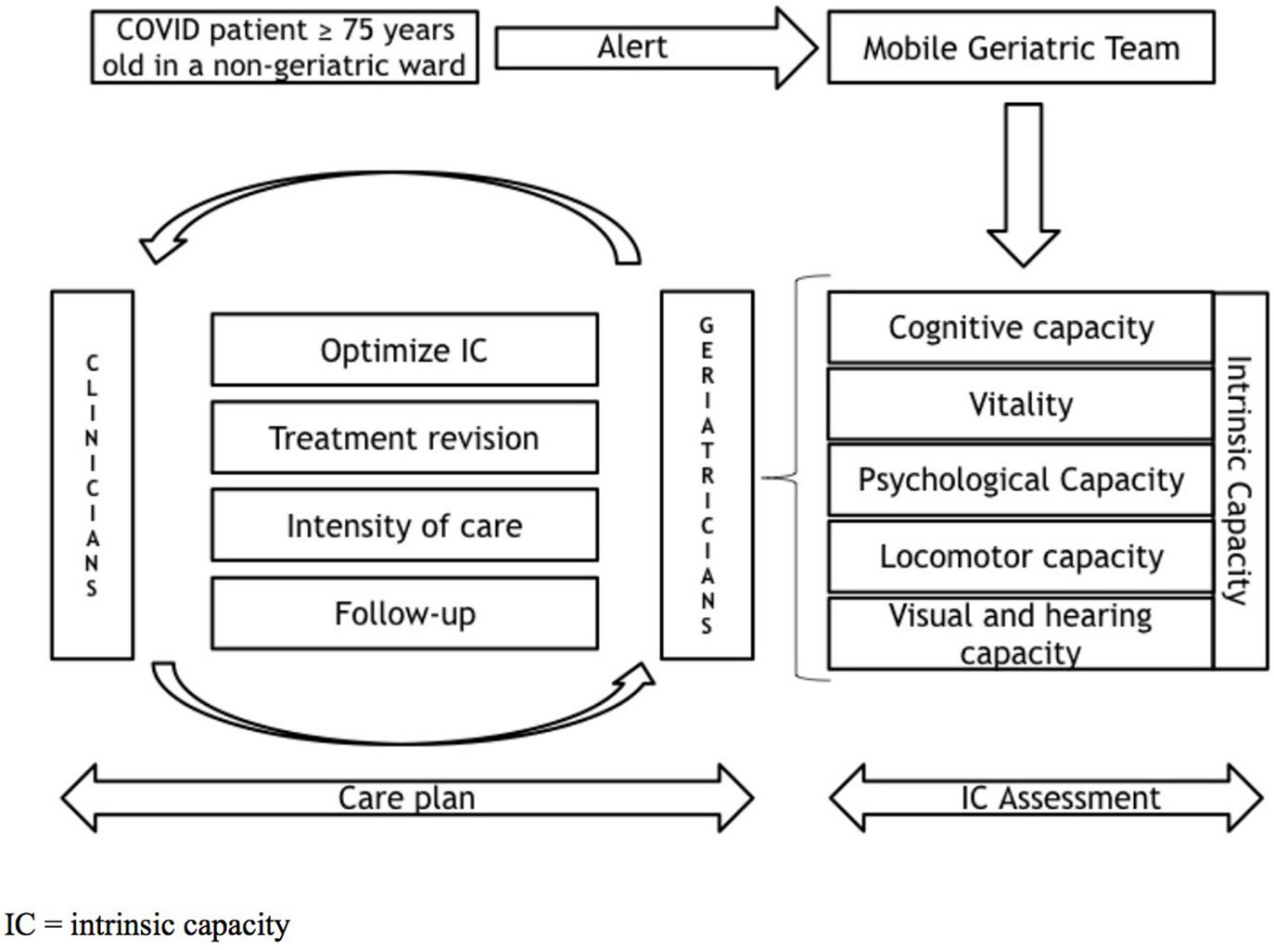
MGT management.

The MGT aimed to systematically assess and optimize intrinsic capacity and support non-geriatrician physicians in implementing an individualized care plan during hospitalization and after hospital discharge (Figure 1). For each patient over 74 years old hospitalized for COVID-19 infection, an alert was generated and managed by the mobile geriatric team nurse coordinator (Figure 1). Because geriatric age is set in Belgium at 75 years old by the Royal Decree defining the standards of geriatric care program and its components, MGT assessed only patients aged 75 years old or older (14).

According to the institutional COVID-19 registry, 557 patients with COVID-19 were hospitalized between October 1st and December 4th, in a non-intensive care unit of our academic hospital of 850 beds, the Erasmus Hospital. The mean age was 66.2 (±11.7) years. Two hundred and two patients were 75 years old or more, and among them, 105 (52%) were hospitalized in COVID geriatric units, while 97 (48%) were oriented to COVID medical and surgical non-geriatric units. Among the patients hospitalized in non-geriatric wards, 49 (51%) were assessed by the MGT.

Forty-eight patients (49%) did not benefit from the mobile geriatric team assessment due to the severity of their clinical status (e.g., hemodynamic instability or respiratory distress, end-of-life status, or not able to answer the questions) or because the patient refused the geriatric assessment. Data were extracted from the “COVID-19 Seniors Registry,” the registry of patients aged 75 years old or older hospitalized for COVID-19 infection in our hospital. The local ethics committee (Comité d'Ethique Hospitalo-Facultaire Erasme-ULB) approved this project on June 25, 2020, under the reference number SRB2020/209—P2020/320.

## Assessment and Optimization of Intrinsic Capacity

The World Health Organization (WHO) published a handbook guidance called the Integrated Care for Older People (ICOPE), whose ambition is to reduce the number of dependent people in the next decades (15). The objective is to promote healthy aging by optimizing intrinsic capacity functions. In order to achieve this goal, ICOPE proposes a program consisting in five actions called steps: the screening of intrinsic capacity decline, the complete assessment of intrinsic capacity functions, the implementation of a personalized care plan, the monitoring of the care plan, and the integration of the caregivers and the community (16). The importance of assessing intrinsic capacity in the context of COVID-19 has been previously discussed and is justified by the strong impact of COVID-19 infection on the key functions of the aging population (17). The present MGT model was inspired by steps 1 (screening of IC decline) and 2 (complete assessment of IC functions) of ICOPE recommendations (Table 1).

**Table 1:** Intrinsic capacity assessment by domain.

**IC domain**	**GMT actor(s)**	**Tool(s)**
Cognition	Nurse—occupational therapist	CAM—MiniCog
Mobility	Physiotherapist	Chair Rise Test—SPPB
Vitality	Dietician	MNA-sf
Psychology	Psychologist	GDS-4
Sensory	Nurse	WHO eye chart—Whisper test

*IC, Intrinsic capacity; MGT, Mobile Geriatric Team; CAM, Confusion Assessment Method; MiniCog, Minimal cognitive assessment tool; SPPB, Short Physical Performance Battery; MNA-sf, Mini Nutritional Assessment short form; GDS-4, Geriatric Depression Scale 4 items; WHO, World Health Organization*.

At the beginning of hospitalization, IC assessment was performed by the different actors of the mobile geriatric team. Every actor discussed his/her workup with the geriatrician, who thereafter delivered MGT recommendations to the clinicians and nurses in charge of the patients orally and by a written note in the patient medical file (accessed by all the members of medical and paramedical team in charge of the patients). During the stay, the MGT continued to follow the patients, the frequency of their interventions varying according to the domain and the clinical situation. During this phase, the MGT actors monitored the evolution, adapted the recommendations proposed, and referred to geriatricians if they considered that a medical advice was needed (e.g., patient who presented a significant weight loss were considered to benefit from a parenteral nutrition). Patients assessed by the MGT were systematically weekly discussed.

### Cognitive Capacity

Delirium is a common condition in older adults admitted for COVID-19 (18) and is associated with high in-hospital mortality (19). Moreover, COVID-19 has been associated with short-term cognitive decline (20). The assessment of cognitive domains focused on the screening and treatment of delirium and a short cognitive assessment. Recommendations to prevent or manage delirium were proposed to clinicians and nurses in charge of the patient from the day of assessment. An occupational therapist assessed autonomy status (upon admission, during hospitalization, and before hospital discharge) and asked the relatives about previous problems of memory, orientation, speech, language, or any difficulties with performing basic and instrumental daily activities.

### Locomotor Capacity

Prolonged bed rest has been associated with poor outcomes in older adults hospitalized for infections (21). Frail patients have better outcomes if they receive exercise therapy during hospitalization (22). High inflammatory and hypercatabolic status owing to COVID-19 infection and bed rest lead to an important reduction of functional performances. This may compromise the recovery of functional capacities and induce loss of autonomy. There is evidence that patients with severe COVID-19 need prolonged exercise therapy to prevent or reverse disability (23). Physical therapists assessed mobility upon admission and proposed in-room individualized programs of exercises using an information leaflet for the patients. Thereafter, the physical therapist followed or adapted this program daily during the stay.

### Vitality

COVID-19 patients present a high risk of malnutrition (24) and sarcopenia (25). A poor nutritional status could contribute to increasing the risk of clinical complications (2). According to the current recommendations, all patients were assessed by a dietician upon admission to choose the best nutritional pathway strategy, which was regularly reassessed during hospitalization. If swallowing disorders were suspected, a speech therapist was consulted.

### Psychological Capacity

During hospitalization, because of distancing with relatives, room isolation, and visiting ban, older people with COVID-19 fell often abandoned, fearful, and sometimes unable to understand the situation. This contributes to the onset of anxiety and depressive disorders (26, 27). If long-term psychological consequences are still unknown, anxiety, and depressive disorders have been associated with a significant cognitive decline risk (20). For patients able to communicate, a psychological support was provided during hospitalization, and video calls with relatives were organized every day (28).

### Sensory Domain

Vision and hearing impairments are common in older adults and have been associated with an increased risk of delirium and higher mortality during hospitalization (29, 30). Furthermore, during the pandemic, the wearing of masks, visors, and social distancing were major obstacles to communication between patients and healthcare providers. In this context, it was recommended to nurses that patients wore hearing devices and glasses as indicated as possible, and to speak them slowly and clearly.

## Care Plan

Geriatricians of the MGT discussed with clinicians about an individualized care plan, taking care not to modify or substitute the routine management of COVID-19 infection, but rather to counsel how to optimize intrinsic capacity as discussed previously, but also how to review medical treatment, define the intensity of care, and organize follow-up (Figure 1). During the COVID-19 pandemic, given the limited number of intensive care beds, a major challenge for healthcare providers was to identify patients who would be the most likely to benefit from intensive care. According to a national survey led by the Belgian Society of Gerontology and Geriatrics in June 2020, one of the most difficult issues for the physicians was the feeling of loneliness while having to make decisions around the intensity of care and the sense of powerlessness in front of a high mortality rate^2^. Although it is known that mortality rate due to COVID-19 increases with age (31), several studies found that chronological age alone is not a good predictor of COVID-19 lethality in individuals without comorbidities or robust (32, 33). Indeed, chronological age alone does not directly reflect the homeostastic and homeodynamic changes making an individual more susceptible to a poor COVID-19 prognosis (34). Geriatricians frequently discussed with clinicians about reasonable limitations of the therapeutic efforts when needed, or at the contrary, to consider ICU admission for older patients with higher resilience. The decision-making was based on the individual's frailty status, comorbidity, and opinions and wishes rather than chronological age per se. Frailty status was assessed by the Clinical Frailty Scale (35), as proposed by different geriatric societies (36–38). Considering comorbidity burden, geriatricians focused on pathologies and geriatric syndromes that have been associated with a poor prognosis in the context of COVID-19, like dementia, type 2 diabetes, or chronic obstructive pulmonary disease (39). Patients able to communicate and understand the situation were questioned on the intensity of care they wished. When this was not possible, geriatricians enquired about existing advanced directives or patients' wishes by discussing with patients' relatives and their general practitioner.

Due to lockdown restrictions, access to ambulatory care was limited to urgent situations (40). Likewise, for COVID-19 patients admitted in healthcare facilities, hospitalization time was almost exclusively allocated to the treatment of COVID-19 infection. For this reason, chronic diseases and new incident diseases were often not optimally managed or were neglected. In this context, a post-discharge plan was proposed, including consultations in geriatric day hospital and/or referral to other specialists. Persons who have severe COVID-19 infection might take several months to return to normal mobility (41). With this in mind, after a careful reviewing of patients' mobility capacity, MGT considered a transfer to a rehabilitation unit at discharge or a home-based individual physical exercise program.

## Conclusion and Perspectives

During the second wave of the COVID-19 pandemic, the admission of geriatric patients in non-geriatric units was widespread and therefore particularly challenging for healthcare providers without geriatric training. In this perspective paper, we proposed the first description of a new approach based on the systematic screening of IC functions by a multidisciplinary mobile geriatric team in a hospital setting. Although we presented a single-center experience, the implementation of this model may promote multidisciplinary management of older adults in non-geriatrics wards and solicit attention to often neglected (but critical) aspects of the individual's health status. By raising awareness about the key functions of the persons, it is possible to obtain a comprehensive assessment of the health status and design adequate interventions for potentially preventing or reversing functional decline, even in emergency situations as the COVID-19 pandemic. Ethical decision-making is a stressful skill task in medical practice and was even more difficult during the COVID-19 crisis. The decision-making process was based on the individual's frailty status, comorbidity burden, and patient's wishes and priorities. The COVID-19 pandemic highlighted the need for markers of resilience capacity in clinical practice. In the future, these markers could be integrated into ethical decision-making algorithms. Further studies are needed to establish if, during the COVID-19 pandemic, a geriatric assessment was beneficial in terms of length of stay and functional, psychological, and cognitive outcomes.

## Data Availability Statement

The raw data supporting the conclusions of this article will be made available by the authors, without undue reservation.

## Ethics Statement

The studies involving human participants were reviewed and approved by Comité d'Ethique Hospitalo-Facultaire Erasme-ULB. Written informed consent for participation was not required for this study in accordance with the national legislation and the institutional requirements.

## Author Contributions

DA and CN conceived the project, drafted the article. RB was responsible for data extraction and analysis. FV was involved in the critical appraisal of the manuscript. MC modified the article with important intellectual content. SD conceived the project, drafted the article, was involved in the critical appraisal of the manuscript. All authors contributed to the article and approved the submitted version.

## Conflict of Interest

The authors declare that the research was conducted in the absence of any commercial or financial relationships that could be construed as a potential conflict of interest.

## Footnotes

^1^Available online at: https://covid-19.sciensano.be/fr/covid-19-situation-epidemiologique (accessed December 12, 2019).

^2^Available online at: https://geriatrie.be/media/2020/10/8.DeBreucker-S-Enquete-COVID-en-geriatrie.pdf (accessed December 12, 2019).

## Abbreviations

COVID: Coronavirus disease
MGT: mobile geriatric team
IC: intrinsic capacity
ICOPE: Integrated Care for Older PEople.
